# FASD and ADHD: Are they related and How?

**DOI:** 10.1186/s12888-016-1028-x

**Published:** 2016-09-22

**Authors:** Larry Burd

**Affiliations:** North Dakota Fetal Alcohol Syndrome Center, University of North Dakota School of Medicine and Health Sciences, 1301 N Columbia Road Stop 9037, Grand Forks, ND 58202-9037 USA

## Abstract

In this issue of the journal, consensus criteria for the diagnosis and management of attention deficit hyperactivity disorder (ADHD) in people who have fetal alcohol spectrum disorders (FASD) are presented. In the absence of an adequate body of research on diagnosis and intervention, this expert consensus opinion is a welcome advance and should provide some guidance for clinicians managing people with FASD who have a comorbid ADHD.

In a previous commentary we posed the question "Treatment of FASD Are We Ready Yet?" [﻿^1^]﻿. One measure of this progress is the developmen﻿t of criteria for identification and treatment of people with ADHD and FASD [2]. ﻿In the United States, 7.3 % of women of child bearing age (3.3 million) are at risk for an alcohol exposed pregnancy [3]. Prenatal alcohol exposure results in about 1 % of live births having an FASD [4]. One measure of the severity of the addiction in these women is the 44 fold increase in their premature mortality [5]. The United States would have about 40,000 new cases of FASD each year. The birth through 18 years of age population, is about 720,000 people. If we utilize the recommended multidisciplinary team model:

- open 50 weeks per year
- 5 days a week
- evaluating four new patients per day
- a screening yield of around 25 %
- a no-show rate of 40 %
- clinic capacity 1,000 evaluations per year

Our 40,000 new cases each year x the 4 cases who need to be seen in order to diagnose one case of FASD, then becomes 160,000 evaluations. When we add the 40 % no-show we are at 224,000 evaluation slots needed each year just for the annual birth cohort. The United States would need to fund another 200 multidisciplinary teams to meet this demand. Using the same variables, the birth through 18 population of 720,000 people requires 3.9 million multidisciplinary FASD evaluations.

If the rates are similar in the United Kingdom the 6,985 FASD cases require 39,116 multidisciplinary evaluations. This would require 39 full time FASD teams for the new FASD cases each year in the United Kingdom. The same problem exists for the birth through 18 population of 125,730 people with FASD.

This is likely an optimistic view of the diagnostic dilemma. The most common identifiable cause of intellectual disability is FASD (relative risk 19 fold) [6]. FASD also appears to be the leading cause of ADHD as well. A diagnosis of FASD is associated with increased risk for ADHD (relative risk = 7.6; attributable risk 86.8 %). Conversely, a diagnosis of ADHD predicts increased risk for FASD (relative risk 13.28; attributable risk 92.5 %). Thus, ADHD and FASD represent an intersection of phenotype expression and complexity. Both disorders have prevalence rates, course, severity, and lifelong impact that suggest they are going to require ongoing assessments and management [6–8]. This would result in even more demand for multidisciplinary FASD evaluations.

However, even improvements in diagnostic capacity may be of limited help in identifying most cases of FASD. The current diagnostic criteria for FASD are so complex that even expert clinicians have difficulty applying them [9]. The barriers around prenatal exposure assessments, assessment of facial features, and brain dysfunction are often developmentally discontinuous and are primarily appropriate for children 2–14 years of age. It seems that at least 90-95 % of all cases of FASD are either undiagnosed or misdiagnosed [9]. This is in agreement with our experience.

What would help? Large numbers of people with FASD (mostly undiagnosed) have developmental disabilities and mental disorders and are cared for by psychiatrists and other mental health clinicians. The recent publication of FASD diagnostic criteria (Neurodevelopmental Disorder associated with Prenatal Alcohol Exposure) in the most recent revision of the Diagnostic and Statistical Manual of Mental Disorders seems to offer the optimal path to making diagnosis more readily available [10]. While the proposed criteria are presented in the appendix of the DSM and are not yet accepted criteria they do overlap very closely with the FASD diagnostic category of alcohol related neurodevelopmental disorder [11, 12]. As a result we now have readily applicable criteria for the most common manifestation of FASD encompassing about 85 % of affected people [11]. The proposed criteria in the DSM are written in a familiar presentation that can readily be used by a wide range of clinicians and researchers who utilize current DSM criteria.

What next? Firstly, improved diagnostic strategies are needed. It is simply not possible for multidisciplinary diagnostic teams to efficiently provide access for: 1) the huge population of people born each year with FASD; 2) the undiagnosed population of children and adolescents; and 3) the much larger population of undiagnosed adults. The solution - use the DSM criteria and make diagnosis available near the treatment services. Some proportion of children and adolescents may need multidisciplinary evaluations and they can be referred out to the existing FASD diagnostic teams. This change would allow diagnosis by community psychiatrists, pediatricians, psychologists, neurologists, and mental health professionals, who are the primary providers of care for people with FASD. Secondly, well designed studies of treatment of ADHD in children with FASD are urgently needed and should be prioritized. Thirdly, since FASD appears to be the most common identifiable cause of ADHD, studies examining the prevention of ADHD in FASD are needed. This could also have an important impact on phenotype severity and decrease the complexity of treatment. Fourthly, the lack of diagnostic and billing nosology’s for FASD also limit research examining cost and health care utilization. In several thousand cases of ADHD our research group fo﻿und almost no cases of FASD in the health claims data. [13, 14]. This is not due to the absence of cases, but rather the limitations of the diagnostic and billing nomenclature for FASD. Since we have an FASD registry with several hundred cases of FASD who also have ADHD, we can estimate the missing proportion of cases and over 90 % of the cases were identified. As a result it’s very difficult to identify FASD accurately in either the diagnostic databases or in the health care cost or utilization databases. Fifthly, we need to begin to figure out how to identify FASD in adults and the elderly. We have people with FASD we have now followed for over 30 years. For many, the problems continue to increase and the toxic effects of prenatal alcohol exposure in the elderly do not resemble the phenotype seen in children and adolescents.

Recommendations 1–4 above support the inclusion of the criteria for Neurodevelopmental Disorder Associated with Prenatal Alcohol Exposure in the next revision of the DSM. The move up front from the back of the book and an upgrade in status as an accepted diagnostic entity should be a public health priority. If the DSM criteria are included in the next revision, it’s likely that more people will be diagnosed with FASD in the next couple of years than have been diagnosed since fetal alcohol syndrome was reported over 40 years ago in 1973 [15]. It seems likely that early diagnosis and early access to treatment could reduce the increasing complexity of the FASD phenotype across the lifespan. These few steps may offer an opportunity to reduce the number of undiagnosed or misdiagnosed people, and may improve access for people with FASD to the appropriate and individualized treatment they require and deserve.

## References

[1] Burd L, Christensen T (2009). Treatment of fetal alcohol spectrum disorders. Are We ready Yet?. J Clin Psychopharmacol.

[2] Young S, Absoud M, Blackburn C, Branney P, Colley B, Farrag E, et al.: Guidelines for identification and treatment of individuals with attention deficit/hyperactivity disorder and associated fetal alcohol spectrum disorders based upon expert consensus. BMC Psychiat, in press. Doi:10.1186/s12888-016-1027-y. 10.1186/s12888-016-1027-y PMC5032241 27655132

[3] Montag AC (2016). Fetal alcohol-spectrum disorders: Identifying at-risk mothers. Int J Wom Health.

[4] Paintner A, Williams A, Burd L (2012). Fetal alcohol spectrum disorders: Implications for child neurology. Part 1: Prenatal exposure and dosimetry. J Child Neurol.

[5] Li Q, Fisher WW, Peng CZ, Williams AD, Burd L (2012). Fetal alcohol spectrum disorders: A population based study of premature mortality rates in the mothers. Mat Child Health.

[6] Popova S, Lange S, Burd L, Rehm J (2012). Health care burden and cost associated with fetal alcohol syndome: Based on official Canadian data. PLoS ONE.

[7] Popova S, Lange S, Burd L, Rehm J: The economic burden of fetal alcohol spectrum disorder in Canada in 2013. Alcohol Alcohol 2015, 1–11 10.1093/alcalc/agv117 26493100

[8] Burd L, Klug MG, Martsolf JT, Kerbeshian J (2003). Fetal alcohol syndrome: neuropsychiatric phenomics. Neurotox and Teratol.

[9] Chasnoff IJ, Wells AM, King L (2015). Misdiagnosis and missed diagnoses in foster and adopted children with prenatal alcohol exposure. Pediatrics.

[10] American Psychiatric Association, 2013. Diagnostic and statistical manual of mental disorders (5th ed.). Washington,DC: Author

[11] Burd L (2016). Invited commentary: FASD: complexity from comorbidity. Lancet.

[12] Stratton KR, Howe CJ, Battaglia FC, Institute of Medicine (1996). Fetal alcohol syndrome-diagnosis, epidemiology, prevention, and treatmen.

[13] Burd L, Klug MG, Coumbe MJ, Kerbeshian J (2003). Children and adolescents with attention deficit-hyperactivity disorder: 1. Prevalence and cost of care. J Child Neurol.

[14] Burd L, Klug MG, Coumbe MJ, Kerbeshian J (2003). The attention deficit-hyperactivity disorder paradox: 2. Phenotypic variability in prevalence and cost of comorbidity. J Child Neurol.

[15] Jones KL, Smith DW (1973). Recognition of the fetal alcohol syndrome in early infancy. Lancet.

